# Health economics and financing for health systems transformation: lessons from five African epistemological frameworks

**DOI:** 10.11604/pamj.2026.53.152.51164

**Published:** 2026-04-10

**Authors:** Alex Olateju Adjagba, Aboubakar Kampo, George Laryea-Adjei

**Affiliations:** 1 Health Economics and Financing Section, UNICEF Global Health Practice, Centre of Excellence, Nairobi, Kenya,; 2 UNICEF Representative, Ethiopia Country Office, Addis Ababa, Ethiopia,; 3 Programme Division, UNICEF Headquarters, New York, USA

**Keywords:** Community-centered health systems, global health governance, health economics, health financing, decolonization, epistemic transformation, African health systems, participatory governance, postcolonial political economy, health systems reform

## Abstract

Persistent financing challenges in African health systems are often attributed to resource scarcity or weak implementation. This paper argues that a deeper structural issue lies in the continued reliance on inherited vertical health architectures and economic evaluation frameworks that remain epistemically misaligned with local social realities. Many contemporary financing models, shaped by colonial and postcolonial donor interventions and operationalized through tools such as disability-adjusted life years (DALYs) and cost-effectiveness thresholds, prioritize externally defined metrics of value. As external funding declines and fiscal pressures intensify, sustaining these imported models exposes tensions between technical efficiency and social legitimacy. This study examines whether reorienting health economics through African epistemological frameworks can improve the sustainability, legitimacy, and governance of health financing. The paper employs a decolonial analytical framework drawing on the work of Ngũgĩ wa Thiong'o, Felwine Sarr, Samir Amin, Valentin Mudimbe, and Paulin Hountondji. It combines historical analysis of colonial health system design, conceptual synthesis of African epistemic frameworks, and applied examination of contemporary financing domains, including insurance uptake, community health worker compensation, and decentralised budgeting. Secondary literature from health policy, political economy, and governance studies is used to assess how epistemic assumptions shape institutional outcomes and financing sustainability. The analysis demonstrates that financing challenges are not solely fiscal but epistemic. Imported models often fail where they conflict with social understandings of care, reciprocity, and collective responsibility, contributing to enrollment gaps, workforce tensions, and performative participation in budgeting. African intellectual frameworks converge in identifying language, valuation systems, and institutional design as sites where power and legitimacy are negotiated. Evidence from insurance reform, workforce governance, and participatory budgeting shows that when financing mechanisms are adapted to locally grounded priorities, they are more likely to generate trust, accountability, and durable engagement. However, risks persist, including elite capture and the symbolic adoption of decolonial language without structural change. Decolonizing health economics and financing requires more than redistributing resources; it demands epistemic and institutional transformation. Aligning financing models with community-defined priorities, plural valuation frameworks, and participatory governance can reduce structural friction and improve legitimacy, even within globally interconnected economies. While such reform will not resolve all fiscal constraints, it offers a pathway toward more socially grounded and sustainable health systems. This reframing, described as a “structural adjustment of the soul”, calls for African-led intellectual and institutional renewal capable of integrating analytical rigor with epistemic legitimacy in health policymaking.

## Introduction

What if the enduring challenges in African health financing stem not only from resource scarcity but also from the persistent influence of inherited vertical programs and legacies of colonial health interventions that remain fundamentally misaligned with African contexts, needs, and priorities [1]? While this analysis focuses primarily on African experiences, it is crucial to recognise that the challenges examined here extend far beyond the continent, encompassing all societies in which colonialism was established to a significant extent. The systematic imposition of external health models, the marginalisation of indigenous knowledge systems, and the creation of dependency relationships in health financing represent universal patterns affecting colonised societies across Latin America, Asia, Africa and the Pacific. The structural adjustment programs of the 1980s and 1990s provide particularly compelling evidence of this universality, as the International Monetary Fund and the World Bank imposed virtually identical health-sector reforms across countries as diverse as Brazil, Chile, the Philippines, Benin, Pakistan, Nigeria, and Kenya. These programs demanded the privatisation of health services, the elimination of government deficit financing, and the commodification of public goods such as healthcare, thereby reproducing colonial patterns of external control over domestic health policy decisions. The current crisis in African health financing reveals deeper structural issues, rooted in colonial legacies, neocolonial plans, and postcolonial dependencies. Many health systems currently operating across Africa were established or heavily influenced by external donors [2]. These systems reflect priorities that served colonial or geopolitical interests rather than community-defined needs. Contemporary health governance continues to reflect these colonial legacies through what scholars term “structural violence”, economic policies that prioritise financial sustainability over access to healthcare in countries where “resource limitations” have always been externally imposed. From Kenya's 2024 Finance Bill protests to Latin American countries' ongoing struggles with World Bank conditionalities, the same epistemological patterns operate across the formerly colonised world [3].

Now, as donor funding diminishes, countries find themselves in debt. They are struggling to maintain these inherited models while fundamental questions about their relevance and sustainability remain unexamined. While broader patterns of epistemological colonisation in global health have been well documented elsewhere, their specific influence on the disciplines of health economics and financing, and how these economic frameworks subsequently shape health system design and outcomes, remains underexplored. Africa thus serves as an analytical case study for examining these economic and financing dimensions of colonial knowledge systems, patterns that demand attention from health economists regardless of their geographic focus. This question is central to current health policies across the African continent and demands more than technical solutions or increased funding. It requires a fundamental rethinking of how we conceptualise, design, and finance health systems. Decolonised frameworks developed by African intellectuals such as Ngũgĩ wa Thiong'o, Samir Amin, Valentin Mudimbe, Paulin Hountondji, and Felwine Sarr offer African governments critical analytical tools for this transformation. These frameworks challenge all stakeholders to move beyond inherited paradigms toward health economics and financing grounded in African epistemologies and community agency. This essay aims to bridge the gap between structural critique and practical transformation. It provides frameworks for African-led health economics and financing that respect both the complexity of inherited challenges and the richness of indigenous solutions. It makes three main contributions: first, it shows how colonial health intervention models continue to influence contemporary financing structures; second, it integrates decolonial theoretical frameworks from African scholars into practical health economics applications; and third, it suggests concrete pathways for implementing community-centred health financing that emphasises African agency and knowledge systems.

## Essay

### Section I: background on health systems and health economics teachings in Africa

#### The colonial architecture of contemporary health financing

The historical development of vertical health interventions reveals a complex legacy, deeply intertwined with colonial structures and policies, that continues to shape African health systems today [4]. The Balmis Expedition is one of the earliest examples of a disease-specific global health campaign [5]. In this campaign, smallpox vaccination efforts were systematically directed and coordinated by the Spanish Empire. These efforts spanned their vast territories, establishing a precedent for centralised, top-down health interventions [5] that would later influence modern global health strategies. Colonial administrations further developed this vertical health infrastructure by establishing sanatoriums in colonial regions, creating specialised facilities focused on the isolation and treatment of specific diseases. This was particularly evident for tuberculosis [6]. These institutions served dual purposes as medical facilities and instruments of colonial health control. They allowed colonial powers to manage disease outbreaks while maintaining administrative oversight over local populations. This approach was particularly evident in the development of Indigenous tuberculosis hospitals. In these facilities, segregated, disease-focused care systems reflected broader racialised colonial policies [7]. The colonial-era foundations of vertical health programming found new expressions in the twentieth century. This occurred through the Rockefeller Foundation's disease-specific intervention model [4]. This foundation-driven approach to global health was modernised in its methods and rhetoric. However, it was fundamentally built on the colonial-era vertical framework established by earlier imperial health campaigns [8]. The Rockefeller Foundation institutionalised the practice of targeting specific diseases through dedicated programs, resources, and infrastructure. This approach created a template that would significantly influence contemporary global health policy and practice.

Today, the fingerprints of this colonial influence remain embedded in contemporary African health systems. The persistence of colonial logics in postcolonial African states, which Achille Mbembe terms the 'postcolony,' demonstrates that independence did not necessarily translate into epistemological freedom in health system design [9]. This influence is reflected in their foundational structures, priority domains, and financing mechanisms. This legacy is evident in the continued dominance of vertical and disease-specific programs. It also appears in external priority-setting and in reliance on imported models. These approaches often fragment care delivery and resource allocation, making it challenging to build horizontal, comprehensive, and integrated health systems. Health agendas continue to be shaped by international organisations, western philanthropies, donor preferences, and global health emergencies. These agendas involve limited meaningful participation from the communities these systems are meant to serve [2] and perpetuate a colonial mindset where African voices remain marginalised in defining their development paths [10,11]. In some cases, African voices choose to remain silent for other motives [10].

Beyond funding questions, the relevance of economic evaluation frameworks is a critical concern. Metrics such as Disability-Adjusted Life Years (DALYs) and cost-effectiveness thresholds developed in Western contexts are applied in Africa. This occurs without a thorough examination of their cultural assumptions or their local relevance [12]. The troubling reality is that many African researchers and policymakers have become so accustomed to these imported frameworks. Few question their appropriateness or explore alternatives, raising fundamental questions about intellectual dependence. These dependencies persist not merely because of resource constraints but also because of insufficient engagement with the question of whether these tools serve African realities. This persistence of colonial architecture ultimately raises fundamental questions about the sustainability and appropriateness of current approaches to health economics and financing. Are we struggling to finance health systems because we are maintaining structures that no longer serve our needs? Could resources currently allocated to sustaining legacy systems be redirected toward more pressing needs, such as providing fair compensation for healthcare workers or implementing community-centred care models?

#### Reclaiming the “Decolonisation” concept itself

Before exploring frameworks for transformation, we need to recognise a troubling irony. The term “decolonisation” has increasingly been co-opted by the very institutions and power structures it aims to challenge. This co-optation is often accompanied by a linguistic shift without accompanying structural transformation. While many international health economics and public health conferences prominently feature “decolonisation initiatives”, these efforts are primarily led, funded, and shaped by Northern institutions, universities, and organisations [13]. This illustrates a sophisticated form of intellectual capture in which the language of liberation is appropriated to uphold existing hierarchies under the guise of a progressive appearance [13]. When “decolonisation” becomes another programme run by those who benefit from colonial structures, it loses its transformative potential. It becomes a tool for legitimising the status quo [9]. This is the emergence of “decolonisation-washing”, in which institutions adopt the terminology while maintaining fundamental power imbalances. Moreover, discussions about decolonisation often become entangled in familiar debates about resource disparities. These include the concentration of research funding in Northern institutions and the limited access of Southern researchers to prestigious journals [14]. While these structural barriers are real and significant, focusing solely on them overlooks a deeper challenge. Would decolonisation be achieved if these resource gaps were eliminated? Consider this scenario: if a major funder provided substantial research funding directly to Southern institutions and leading journals, and offered unlimited access to articles from these grants, would we see a genuine transformation? The answer is not so straightforward. Without fundamental changes in how research is conceptualised, conducted, and evaluated, we are likely to see more of the same methodological approaches, simply dressed in different institutional banners.

We do not entirely dismiss the weight of the existing resource disparity. However, true transformation requires more than just redistributing resources or rearranging institutions [15]. It requires a clear, practical definition: genuine decolonisation must involve epistemological change led by, accountable to, and primarily benefiting those who have been colonized [16]. This includes not only African-led initiatives and African-controlled resources. It also covers African-reimagined methods, African-contextualised metrics, and African-decided measures of success. The main question for all stakeholders, both North and South, is whether we are ready to undertake this deeper transformation. For decolonisation to have a meaningful impact, it must be approached with both cultural and epistemological humility. This means that scholars, project coordinators, and allies involved in decolonial efforts should prioritise greater sensitivity towards communities affected by colonisation. This is especially vital for those often excluded from mainstream conversations. The time has come to decolonise the concept of “decolonisation” itself. We must ensure it maintains its radical potential rather than becoming merely another tool of intellectual domination. This requires vigilance about who speaks for decolonisation, who funds it, who controls its narrative, and who ultimately benefits from its implementation.

### Section II: central frameworks from African intellectuals and recent developments

#### Learning from Ngũgĩ wa Thiong'o: language, knowledge and power

Thiong'o's seminal work on decolonising the mind [17] offers vital insights for transforming health economics and financing. His argument that liberation begins with reclaiming language, culture, and the right to define our realities goes well beyond linguistics. This framework challenges fundamental structures across all sectors, including healthcare. Applying Ngũgĩ's decolonial perspective to health economics and financing uncovers how language and framing influence policy and practice. Terms such as “efficiency,” “burden,” and “dependence” embody colonial assumptions that shape how health issues are understood and managed. The dominance of English and other colonial languages in health policy development can estrange African professionals from their real-world contexts. This linguistic supremacy treats communities as data points rather than as active partners in the search for solutions. African health systems remain largely operational in colonial languages, hindering the growth of research and theory rooted in indigenous conceptual frameworks. Ngũgĩ's concept of endogenous teleonomy offers a different path for health economics practice and resource allocation. This idea suggests that African societies should define their own health system goals and developmental trajectories [18]. When translated to health economics, this approach fundamentally transforms how we conduct economic evaluations, prioritise interventions, and allocate scarce health resources. In the area of economic evaluation methodologies, rather than applying Western cost-effectiveness thresholds (typically $1-3 times GDP per capita), endogenous approaches would develop locally-derived willingness-to-pay thresholds based on community values and priorities. This means incorporating traditional healing costs and community health outcomes that standard DALYs fail to capture, such as social cohesion and spiritual wellbeing [19].

In the area of transforming budget advocacy and resource mobilisation, this would mean a transparent budgeting that prioritises domestic resource mobilisation over donor dependency, reflecting community-defined health priorities rather than externally imposed disease-specific targets [20]. Research demonstrates that approximately 80% of people in developing countries use traditional medicine [21], yet health budgets typically allocate less than 3% to traditional medicine systems. Endogenous budgeting would reverse these priorities, allocating resources proportional to actual community usage patterns. Transparent budgeting and domestic resource mobilisation would serve as expressions of sovereignty. They would reflect community-defined health priorities rather than externally imposed agendas. This approach requires moving beyond the colonial mentality that views Western biomedical knowledge as the only legitimate form of healing. In the redesign of health financing mechanisms, African traditional knowledge systems offer holistic approaches that view health as a balance among physical, spiritual, and emotional well-being. These approaches propose alternative financing models, such as community health funds, that operate through reciprocal obligations rather than individual premium payments. This involves moving beyond colonial economic assumptions that individual rational choice drives health-seeking behaviour, towards community-based risk-pooling mechanisms that mirror African social structures [22]. These approaches stand in contrast to the biomedical model by incorporating community and cultural connections.

#### Learning from Felwine Sarr: afrotopia and economic decolonization

Senegalese economist and philosopher Felwine Sarr broadens these decolonial insights into economic theory. Through his concept of Afrotopia [23], he presents a vision for rethinking Africa's development using culturally rooted, decolonised frameworks. Sarr's work offers specific pathways for transforming health economics. According to Sarr, de-westernising economic thought involves challenging the dominance of Western economic paradigms. He champions approaches that incorporate African values and knowledge systems. In health economics, this results in several key shifts. First, it means prioritising community-based health systems over purely market-driven models. Second, it involves questioning metrics that ignore social cohesion, cultural well-being, and spiritual health. Sarr's framework also emphasises developing accessible ways to explain complex economic concepts. Cost-effectiveness ratios, for example, must be communicated to communities and policymakers who lack economic backgrounds. This represents a critical test of whether our analytical tools truly serve local contexts. Finally, Sarr challenges both Northern and Southern institutions to move beyond resource redistribution. Instead, they must pursue genuine methodological transformation. Sarr introduces an important factor often overlooked by economists: the cultural and symbolic dimensions of health. He recognises that economic interactions are inherently social and symbolic. Sarr's framework emphasises how religious and cultural beliefs shape health behaviours and access patterns. He justifies the need for health interventions that respect local customs and communal decision-making processes. Sarr expands the understanding of health to include multiple dimensions. This incorporates biological, spiritual, and communal balance. This approach contrasts with focusing solely on clinical outcomes. African traditional approaches regard health as an integrated state of balance that encompasses the self, the cosmic realm, and the social world. Health and healing processes that involve loved ones and spiritual values often lead to recovery. Many African cultures define well-being within frameworks of group norms, values, kinship ties, and cultural principles. This holistic view contrasts with the biomedical model by viewing health not merely as the absence of disease, but as a harmony of physical, spiritual, and emotional well-being, connected to community and land. These approaches offer powerful frameworks for fostering positive health outcomes by strengthening cultural identity and social cohesion and addressing the root causes of illness.

#### Samir Amin's structural analysis: delinking and autocentric development

Between Ngũgĩ's cultural decolonisation and Sarr's contemporary reimagining, there lies the foundational economic analysis of Samir Amin [24]. The Egyptian economist provided the structural blueprint for understanding how colonial economic relationships continue in the postcolonial era. Amin, who coined the term “Eurocentrism” [25] and served as the first Executive Secretary of the Council for the Development of Social Science Research in Africa (CODESRIA), offers crucial insights into transforming health economics. His concepts of delinking [25,26] and autocentric development offer frameworks for health system transformation. Amin's central argument was that so-called 'underdeveloped' economies should not be regarded as independent units. Instead, they serve as building blocks of a capitalist world economy, where 'poor' nations form the 'periphery', compelled into perpetual structural adjustment concerning the reproduction dynamics of the 'centres' [26]. Applied to health systems´ financing, this analysis reveals how many African countries remain caught in dependency relationships. These countries support health programmes designed elsewhere, use metrics developed elsewhere, and prioritise needs identified elsewhere. In contrast, local health needs and knowledge systems remain marginalised. Amin's solution was “delinking”. This concept does not imply complete autarky but rather the subordination of global relations to domestic development priorities. Delinking creates “autocentric” development, in which the value of each country's output should be set so that agricultural and industrial workers are paid according to their contributions to society's net output. For health economics, this framework suggests several pathways. It refers to endogenous innovation that leads to investment in local health solutions. It involves research on traditional medicine and context-appropriate technologies. It requires controlling financial transactions and trade to influence health system development. Amin estimated that even 70% delinking would represent a significant achievement. He recognised the practical challenges of complete independence. This pragmatic approach offers hope for health systems seeking to balance global cooperation with local sovereignty. It allows the maintenance of beneficial international partnerships while asserting primary accountability to domestic populations [26]. Since Samir Amin's pioneering work on delinking and Eurocentrism, a robust body of scholarship has emerged. Contemporary scholars have provided concrete frameworks for decolonising economics and health policy. His work demonstrates how delinking from imperial systems connects to sustainable development models rooted in local knowledge systems. Meanwhile, Sabelo Ndlovu-Gatsheni has bridged Amin's Marxist political economy with decolonial epistemology [27]. His work demonstrates that Amin's critique of Eurocentrism offers tools for dismantling colonial knowledge systems. These systems continue to shape economic and health policies across Africa. This collective scholarship now provides substantial theoretical and practical resources that economists, health policy experts, and development practitioners can draw on for moving beyond colonial approaches. As Kvangraven argues, this body of work confirms that “if you want decolonisation, go to the economics of Samir Amin” [27].

#### Valentin Mudimbe's analytical framework: deconstructing colonial knowledge systems in health economics and financing

Valentin Mudimbe's seminal work “The Invention of Africa” [28], provides essential analytical tools for understanding how colonial discourse has shaped African identities and knowledge systems. It offers vital insights into the genealogy of Western representations that continue to influence contemporary minds, education, and policymaking narratives in health economics and financing. Mudimbe's deconstructive methodology reveals how colonial epistemological frameworks have consistently positioned Western knowledge as universal while relegating African ways of knowing to the realm of the traditional or the pre-scientific [9]. Building on Mudimbe's analysis, Achille Mbembe's concept of the 'postcolony' further demonstrates how colonial knowledge hierarchies endure in contemporary African institutions, including health systems that continue to operate according to external logics despite political independence [9]. Mudimbe's analysis of how colonial psychiatry [29] pathologised African ways of being and knowing offers a particularly powerful framework for decolonising health policymaking by exposing how Western medical categories have been imposed to delegitimise indigenous healing practices and community-based understandings of wellness. This insight directly applies to health economics, where cost-effectiveness analyses often dismiss traditional medicine as “inefficient” or community health approaches as “unscalable” without scrutinising the cultural assumptions underpinning these judgments. Mudimbe's work shows that such dismissals are not objective evaluations but reflect colonial knowledge hierarchies that persist within current health financing frameworks [29]. When applied to health economics, this analysis shows that seemingly neutral economic concepts such as efficiency, rationality, and optimisation contain embedded colonial assumptions about what constitutes legitimate knowledge and appropriate decision-making processes in health systems [30]. By using his deconstructive approach, health economists can start to see how their disciplinary tools participate in ongoing processes of epistemological colonisation, thereby opening space for the development of culturally grounded and epistemologically diverse approaches to health system design that prioritise African knowledge systems rather than simply adapting Western economic models to local contexts.

#### Paulin Hountondji's methodological reconstruction: rebuilding indigenous intellectual foundations

Paulin Hountondji's “African Philosophy: Myth and Reality” [31,32] presents a rigorous critique of how knowledge systems have been systematically categorised and marginalised through colonial academic structures, offering methodological approaches essential for reconstructing indigenous intellectual traditions within health economics education and practice. Hountondji's work challenges the false dichotomy between “Western science” and “African tradition” by demonstrating that this binary obscures the dynamic, evolving nature of African intellectual traditions and their capacity to engage with contemporary challenges on their own terms. His methodological framework provides health economists with tools for recognising how current global health financing approaches perpetuate these false dichotomies by positioning community-based health financing mechanisms as “informal” or “traditional” alternatives to “modern” insurance schemes, rather than as legitimate knowledge systems with their own sophisticated understanding of risk, solidarity, and resource allocation. Hountondji's pedagogical approaches can be directly applied to reconstruct the epistemic foundations underpinning current global health financing frameworks by encouraging health economists to engage seriously with indigenous concepts of health, healing, and community responsibility as starting points for economic analysis rather than as obstacles to overcome. His emphasis on the collective nature of African intellectual production challenges the individualistic assumptions embedded in Western economic theory. His framework suggests alternative approaches to health system governance that prioritise community decision-making processes over technocratic optimisation. For health economics training, Hountondji's work offers a methodological blueprint for moving beyond simply including “local content” in Western-designed curricula. It advocates for a fundamental rethinking of how economic analysis is conceived, conducted, and applied within African health systems. This involves recognising that concepts like reciprocity, ubuntu, and collective responsibility are not merely cultural artefacts to be fitted into Western economic frameworks. Instead, they serve as nuanced philosophical foundations for alternative approaches to health financing that may be more suitable and effective in African contexts than imported models designed for different social, cultural, and economic realities.

#### Converging African frameworks for the epistemological transformation of health economics and financing

The five theoretical frameworks analysed earlier in this essay converge on the key realisation that transforming health economics in Africa requires more than merely technical adjustments to Western models. These frameworks collectively show that the current crisis in African health systems does not simply arise from resource shortages or implementation issues, but from the deeper epistemological violence of imposing foreign knowledge systems that fundamentally misunderstand African realities of health, healing, and community wellbeing. The convergence begins with Ngũgĩ's insight that language shapes thought, which resonates with Mudimbe's analysis of how colonial discourse constructed African identities and Hountondji's critique of how knowledge systems have been systematically marginalised. Together, these frameworks reveal that health economics terminology itself, concepts like “efficiency,” “cost-effectiveness,” and “rational choice” carry embedded assumptions about human nature, social organisation, and the purpose of health systems that may be fundamentally incompatible with African philosophical traditions emphasising ubuntu, reciprocity, and collective responsibility. This linguistic and epistemological dimension connects directly to Sarr's call for new symbolic universes and to Amin's argument for intellectual independence, suggesting that transforming health economics requires not only policy reforms but also a fundamental reimagining of how economic analysis is conceived and conducted. Amin's structural analysis provides the economic framework for this transformation through delinking from dependency relationships, while Sarr's cultural creativity offers pathways for imagining alternative approaches rooted in African experiences. Mudimbe's deconstructive methodology exposes how seemingly neutral economic concepts perpetuate colonial knowledge hierarchies, while Hountondji's emphasis on collective intellectual production challenges the individualistic assumptions embedded in Western economic theory. The convergence of these five frameworks demonstrates that genuine transformation requires moving beyond superficial modifications to Western models toward the development of genuinely African approaches to health economics that honour both analytical rigour and cultural authenticity.

#### Cross-regional convergences in the epistemic reimagining of health economics and financing

As mentioned in the introduction, the epistemological critique articulated by these African frameworks is neither isolated nor uniquely African; it converges with parallel analyses emerging from other formerly colonised regions where health economics and financing have likewise been scrutinised as carriers of imposed values and power. Scholars examining the global institutionalisation of valuation tools such as disability-adjusted life years (DALYs) demonstrate how ostensibly universal metrics embed historically situated assumptions about what counts as health, whose lives are prioritised, and which forms of knowledge are legitimate, revealing their role as political as well as technical instruments [33]. In Indigenous policy and evaluation traditions in Aotearoa/New Zealand and Australia, researchers similarly argue that mainstream economic evaluation frameworks inadequately capture relational, collective, and holistic understandings of wellbeing, prompting the development of health governance and commissioning models grounded explicitly in Indigenous epistemologies and social accountability structures. Parallel movements in Latin America draw on Indigenous philosophies such as Buen Vivir and Sumak Kawsay to challenge narrow biomedical and market-centred framings of development, advancing culturally situated definitions of health tied to community, territory, and dignity [34]. Across these contexts, calls to decolonise health governance increasingly emphasise the need to rebalance funding authority and decision-making towards locally led institutions, reinforcing the broader argument that transforming health economics is fundamentally an epistemic project concerned with justice, legitimacy, and self-determination rather than merely technical optimisation [14]. Together, these cross-regional perspectives affirm that the African frameworks outlined above participate in a wider global movement to rework the languages, metrics, and financing logics of health so they reflect the lived realities and value systems of communities historically viewed as objects of policy rather than its authors.

#### From emerging progress to structural constraints

The five African frameworks demonstrate that epistemic reorientation in health economics is not merely aspirational; meaningful efforts to embed locally grounded values and governance principles are already emerging. Yet these developments remain structurally fragile. Many initiatives continue to operate within funding architectures shaped by external priorities, thereby constraining domestic autonomy and limiting governments´ capacity to sustain reforms aligned with locally defined health needs. These financial dependencies are compounded by deeper governance challenges. Programs frequently promoted as “community-centred” are often designed externally and implemented through institutional arrangements that concentrate decision-making authority among urban professionals or politically connected actors, creating conditions ripe for elite capture and symbolic participation rather than genuine community agency [35]. Such patterns illustrate how alternative models can be absorbed into existing power structures without producing the structural transformation envisioned by the epistemic frameworks outlined earlier. Addressing these limitations, therefore, requires more than incremental programmatic adjustments. Evidence from global health governance research highlights that sustainable reform depends on strengthening country ownership, redistributing decision-making authority, and aligning financing systems with domestic institutional realities [36]. In African contexts, this involves cultivating domestic leadership and fiscal strategies that support locally controlled development, with health economics tools serving not as externally imposed templates but as adaptable instruments to expand fiscal space and reinforce autonomy [37-39]. These dynamics indicate the need for deliberate structural pathways that translate epistemic transformation into durable governance and national financing reforms, a challenge that frames the practical agenda explored in the following section.

### Section III- Pathways forward: operationalizing epistemic transformation in health economics and financing

#### Principles for transforming health economics

Reimagining health economics through African epistemologies is not merely a symbolic act of diversification but a fundamental shift in how value, responsibility, and decision-making are understood. The earlier outlined theoretical convergence suggests that health systems cannot be sustainably reformed solely through imported models; instead, they must be rooted in locally meaningful notions of wellbeing, reciprocity, and collective duty. This transition requires recognising multiple valuation frameworks, integrating community authority into resource decisions, and tailoring economic tools to mirror social realities rather than enforcing universal metrics. In this perspective, health economics becomes not just a technical discipline of optimization, but a relational practice focused on maintaining social balance, legitimacy, and shared flourishing. These principles lay the groundwork for institutional pathways that can transform epistemic change into governance and financial reforms.

#### Educational transformation

Structural change begins with how health economists are trained. Current curricula frequently present Western analytical frameworks as neutral universals rather than historically situated tools shaped by specific philosophical assumptions. A transformed educational model would therefore treat epistemological context as foundational rather than supplementary. In addition to conventional economic theory, students should engage systematically with African philosophical traditions that foreground reciprocity, relational wellbeing, and collective responsibility, enabling them to interrogate the cultural assumptions embedded in dominant valuation models. This requires incorporating, as core reading rather than elective enrichment, the works of African thinkers whose analyses illuminate the historical, linguistic, and epistemic dimensions of development and governance. Foundational texts by several African scholars provide critical frameworks for understanding how knowledge systems shape policy reasoning and institutional design. Complementary historical modules examining colonial political economy, indigenous governance systems, and the evolution of African social institutions further situate health economics within the lived trajectories of the societies it seeks to serve. Such curricular reform is not a rejection of analytical rigor but an expansion of intellectual scope, preparing practitioners to critically adapt economic tools in ways that resonate with local epistemologies and social realities rather than merely replicating imported templates.

#### Governance and financing reform: adaptive tools for local autonomy

Institutional autonomy is equally essential. Sustainable transformation requires financing structures that prioritise domestic agenda-setting, participatory decision-making, and accountability to communities rather than external funders. Health economics tools, fiscal space analysis, priority-setting frameworks, and budgeting models are increasingly being adapted to support locally defined goals rather than imposed benchmarks. For example, Ghana´s use of fiscal space analysis to inform the expansion of the National Health Insurance explicitly linked economic modelling to domestic political priorities and social protection objectives, demonstrating how technical tools can be embedded within nationally negotiated agendas [37]. In Thailand, participatory priority-setting processes integrated health technology assessment with deliberative social values, offering a model for aligning economic evaluation with legitimacy and public accountability [40]. Similarly, decentralised participatory budgeting experiments in Brazil have shown how fiscal decision-making can incorporate community-defined wellbeing priorities alongside efficiency considerations, thereby reshaping resource allocation through democratic negotiation. When embedded within governance systems that value social legitimacy and collective responsibility, such adaptive uses of economic tools reinforce rather than undermine epistemic autonomy.

#### Implementation ecosystems

As noted at the beginning of this essay, the nature of our implementation systems may partially explain our financing and economic struggles. Reform must extend beyond policy design to the environment in which programmes are implemented. Implementation becomes sustainable when it is rooted in locally embedded institutions rather than externally managed delivery structures. Several experiences in health systems show how aligning execution with social realities enhances legitimacy and effectiveness. Ethiopia´s Health Extension Program, for example, established a locally recruited workforce integrated into community governance structures, facilitating health promotion and preventive care through trusted social relationships rather than distant bureaucratic channels [41]. Similarly, Rwanda´s integration of community health workers into national service delivery combined formal financing with local accountability mechanisms, thereby strengthening collective responsibility for health outcomes [42]. In South Africa, collaborations between biomedical providers and traditional healers have demonstrated how recognising plural knowledge systems can enhance service uptake and cultural legitimacy, especially in HIV and mental health care [43]. These cases show that when implementation ecosystems invest in locally rooted workforces, acknowledge community authority, and incorporate culturally grounded care practices, policies function less as externally imposed programs and more as socially embedded institutions sustained by trust, reciprocity, and shared ownership.

### Section IV- Applied reimagining: concrete illustrations of epistemic translation

#### Reframing health insurance through solidarity logic

In dominant health economics models, insurance is conceptualised as an actuarial mechanism for managing individual risk through advance prepayment. Yet in many African contexts, engagement with formal insurance is shaped by the intersection of economic realities and culturally grounded understandings of risk and wellbeing. In Kenya, for example, the National Hospital Insurance Fund has struggled to extend coverage among informal-sector workers, in part because annual premium structures are poorly aligned with the income volatility of daily wage earners, resulting in low uptake and retention that technical enrollment reforms alone have not resolved [44]. Similarly, Ghana´s National Health Insurance Scheme, despite its explicitly pro-poor design, faces persistent challenges with renewals: wealthier and urban populations are more likely to maintain coverage, whereas poorer groups disengage due to premium burdens, administrative barriers, and uneven access to information. Broader demographic evidence across sub-Saharan Africa confirms that insurance participation remains stratified by socioeconomic status, underscoring that enrollment behaviour reflects both structural constraints and social interpretations of financial risk [45]. Importantly, qualitative research also documents that in some communities, advance payment for illness is perceived as symbolically problematic, sometimes interpreted as risking or “inviting” misfortune, revealing alternative philosophies of uncertainty, reciprocity, and collective care that complicate purely actuarial framings of insurance. From this perspective, low enrollment and renewal should not be dismissed as irrational behaviour but understood as signals that prevailing financing models may not fully resonate with lived social logics. This recognition underscores an urgent need for expanded African scholarship capable of rethinking insurance design through solidarity-based and culturally grounded frameworks, rather than if imported prepayment models are universal solutions.

#### Reimagining community health worker compensation

Conventional debates over community health worker (CHW) compensation frequently frame the issue as a technical problem of payroll sustainability, focusing on whether governments should institutionalise CHWs as salaried cadres or rely on performance-based incentives. While fair remuneration is essential, these framing risks overlook the social foundations that make CHWs effective in the first place. A large body of implementation and ethnographic research demonstrates that CHWs derive their authority not primarily from formal employment status but from embedded community relationships characterised by trust, reciprocity, and social accountability [41,46]. Their work is often understood locally as collective care rather than market labour, with legitimacy deriving from community recognition and relational obligation. Evidence from across African contexts suggests that CHW motivation is sustained by a hybrid economy of modest financial support, social status, and moral commitment, underscoring the limits of purely economic incentive frameworks. Attempts to institutionalise CHWs through payroll-centred or commercialised models reveal important tensions when these social dimensions are neglected. Mozambique´s early donor-supported CHW programs, for example, experienced instability when financing proved inconsistent, leading to compensation interruptions and declining morale, a reminder that adding cadres to payroll without a durable fiscal commitment can undermine program continuity [47]. South Africa´s efforts to formalise community caregivers similarly generated disputes over employment status and funding reliability, highlighting the structural difficulty of integrating large CHW cadres into constrained public budgets [48]. At the same time, ethnographic work from Ethiopia shows that monetisation and entrepreneurial compensation models can reshape perceptions of CHWs, sometimes creating tension between their identity as community caregivers and expectations associated with wage labour or commercial exchange [47]. Broader synthesis research suggests that heavy reliance on financial incentives may crowd out relational motivations and alter trust-based dynamics that underpin CHW effectiveness [46]. These experiences do not argue against compensation; rather, they demonstrate that care work functions as a socially embedded institution whose effectiveness depends on legitimacy within local moral economies. Financing models that treat CHWs purely as labour inputs risk eroding the trust and reciprocity that sustain community engagement. From an epistemological perspective, this evidence underscores that CHW compensation cannot be resolved through technical payroll design alone. Sustainable approaches must recognise the cultural currency of caregiving, the social authority, reciprocity, and collective responsibility that communities attach to health work, and incorporate these dimensions into financing frameworks. In this sense, the challenge is not simply how to pay CHWs, but how to design compensation systems that align with the social ontology of care, illustrating why epistemically grounded health economics is essential for durable workforce policy.

#### Reimagining decentralised budgeting through epistemic lenses

The implications of epistemic transformation extend beyond service delivery into core governance processes such as decentralised budgeting. Across many African countries, decentralisation reforms now mandate community participation in planning and budget formulation, creating the appearance of locally driven priority setting. Kenya illustrates both the promise and the limits of this model. County planning and budgeting processes are structured procedurally by budget circulars, fixed calendars, and technical ceilings that often narrow the space for community priorities to meaningfully shape allocations, even when public participation is formally conducted. Civil society and transparency assessments similarly find that, while countries increasingly report engaging in participation, the depth of information disclosure and feedback loops remains uneven, reinforcing concerns that participation can become performative rather than decisional. Yet there is also evidence that participatory budgeting can be consequential under certain institutional conditions. Research from Brazil´s well-documented participatory budgeting reforms demonstrates that when community deliberation is embedded in decision authority rather than treated as consultation, substantial portions of development resources can be shaped by citizen priorities, with measurable effects on accountability and public trust [49]. Comparable dynamics have been observed in African decentralised contexts. Studies of municipal planning processes in South Africa [50] show that participatory structures can influence allocation decisions when institutional safeguards limit elite brokerage and ensure transparency, while Ghanaian district-level budgeting research highlights cases in which community engagement has meaningfully redirected local investment priorities despite fiscal constraints. These experiences suggest that participatory budgeting is not inherently symbolic; its effectiveness depends on whether institutional design allows locally grounded knowledge to influence fiscal outcomes rather than merely fulfilling procedural requirements.

These patterns show why budgeting is often structurally opposed to epistemic shift: national public financial management systems typically privilege standardised categories, compliance logics, and top-down control (often reinforced through conditional transfers, centrally defined ceilings, and performance reporting), which incentivise “looks-like” participation and limit local epistemic authority over what counts as need and value. The result can be a form of fiscal isomorphism, reforms that mimic participatory ideals while leaving underlying decision power intact. From an epistemological perspective (and consistent with Ngũgĩ´s argument about the politics of language), the core issue is that communities are often forced to translate lived priorities into bureaucratically legible terms that fit predefined templates; what does not fit the template (social cohesion, culturally grounded wellbeing, relational care economies, locally defined vulnerability) risks being excluded by design. An epistemically informed approach would therefore target specific failure points in decentralised budgeting: translation loss (community priorities distorted by administrative categories), fiscal gatekeeping (ceilings and conditionalities that pre-empt deliberation), elite brokerage (local political actors controlling whose voice enters the budget), and feedback collapse (weak mechanisms for explaining why community priorities were accepted or rejected). The practical task is not merely “more participation” but the redesign of budgeting processes so that plural forms of knowledge can enter priority-setting on equal footing through iterative, community-led priority mapping, transparent negotiation of trade-offs under real fiscal constraints, and accountability mechanisms that reward legitimacy and responsiveness alongside technical efficiency.

## Conclusion

This essay started with a key question: are the ongoing difficulties in financing African health systems partly due to efforts to uphold inherited vertical models that were never suited to local realities? The evidence discussed here indicates that, at least in part, the answer is yes. Colonial-era structures and externally driven financing principles continue to influence how priorities are set, resources are distributed, and success is evaluated. When health systems operate within frameworks that misinterpret social understandings of care, risk, and collective wellbeing, financial challenges become structural rather than simply fiscal. Maintaining models that lack epistemic and social legitimacy entails hidden costs, fragmentation, inefficiency, disengagement, and erosion of trust, thereby increasing the financial burden. The convergence of African intellectual traditions articulated by Ngũgĩ wa Thiong´o, Felwine Sarr, Samir Amin, Valentin Mudimbe, and Paulin Hountondji provides a coherent framework for understanding why this misalignment persists and how it might be addressed. The practical cases explored in this essay demonstrate that financing failures often arise where economic models overlook the relational and cultural foundations of governance. These are not isolated technical shortcomings but signals that health economics must consider plural conceptions of value embedded in social life. Redesigning financing systems around epistemically grounded principles is therefore not a retreat from global engagement nor a call for economic autarky. African health systems operate within an interconnected world and will continue to do so. The argument advanced here is more modest and more practical: aligning financing mechanisms with locally meaningful frameworks can improve legitimacy, institutional trust, and social ownership, conditions that are essential for sustainable funding and effective governance. While such a transformation will not resolve all fiscal constraints or eliminate structural inequalities, it can reduce the friction that arises when imported models clash with lived realities. Importantly, epistemic realignment has moral and institutional consequences. Systems seen as socially grounded and responsible are more likely to build public trust, collective responsibility, and resist practices that compromise integrity. Financing is not just about balancing budgets; it also involves maintaining relationships between institutions and the communities they serve. By integrating economic reasoning within culturally meaningful frameworks, health systems achieve not only technical coherence but also ethical legitimacy. The theoretical groundwork for this transformation exists, and early examples suggest that elements of change are already underway. The task ahead is to translate these insights into methodologies, training systems, and governance reforms that recognise health financing as both an economic and epistemological project. Ultimately, enabling African scholars, practitioners, and communities to shape their own economic reasoning is not a promise of perfect systems, but a necessary step toward health institutions that are more durable, equitable, and socially trusted. In this sense, rethinking health economics is not merely a technical adjustment; it is a structural adjustment of the soul of policymaking in Africa.

**Epilogue: language as epistemic infrastructure:** the argument presented throughout this essay ultimately reverts to a question that Ngũgĩ wa Thiong´o placed at the heart of decolonial thought: the power of language to shape how realities are understood and acted upon. Shortly before writing this essay, I came across a research article by an emerging scholar analysing how the “international community” might address ongoing challenges in her country. The analysis itself was rigorous, yet the terminology revealed something deeper. Describing identical geopolitical relationships with labels such as “global South,” “third world,” or “international community” subtly redistributes agency and authority. The words did not merely describe power relations; they reproduced them. This observation highlights Ngũgĩ´s insistence that language is not neutral. Terminology shapes the boundaries of imagination, affecting how problems are framed and what solutions seem feasible. Throughout this essay, familiar divisions such as “North” and “South” were invoked almost instinctively. In other contexts, these same actors are recast as “the West” and “the rest,” or as representatives of an “international community.” Each formulation carries implicit assumptions about legitimacy, hierarchy, and voice that influence policy reasoning, including in health economics and financing. Understanding the epistemic power of language explains why transforming health systems is linked to changing how realities are named and interpreted. Institutional reform without linguistic awareness risks just recreating the hierarchies it aims to dismantle. The task, therefore, is not only to redesign financing mechanisms but also to develop analytical vocabularies that can express locally grounded priorities and social logics. Ngũgĩ wa Thiong´o´s work reminds us that the ability to define our circumstances is inseparable from our capacity to change them. The ongoing rethinking of health economics across Africa is part of this wider epistemic shift. Whether this transformation occurs passively or through intentional intellectual leadership remains an open question. The opportunity and responsibility to shape it lie with those willing to examine not only institutions and budgets but also the language through which futures are envisioned.
